# The Bidirectional Relationship between Weight Gain and Cognitive Function in First-Episode Schizophrenia: A Longitudinal Study in China

**DOI:** 10.3390/brainsci14040310

**Published:** 2024-03-26

**Authors:** Ke Ma, Tianhang Zhou, Chengcheng Pu, Zhang Cheng, Xue Han, Lei Yang, Xin Yu

**Affiliations:** 1 Department of Clinical Psychology, Beijing Chao-Yang Hospital, Capital Medical University, Beijing 100020, China; 2 Peking University Sixth Hospital, Beijing 100191, China; 3 Institute of Mental Health, Peking University Sixth Hospital, Beijing 100191, China; 4 NHC Key Laboratory of Mental Health, Peking University, Beijing 100191, China; 5 National Clinical Research Center for Mental Disorders, Peking University Sixth Hospital, Beijing 100191, China

**Keywords:** schizophrenia, weight gain, cognitive function, first episode, China

## Abstract

Patients with schizophrenia often encounter notable weight gain during their illness, heightening the risk of metabolic diseases. While previous studies have noted a correlation between obesity and cognitive impairment in schizophrenia, many were cross-sectional, posing challenges in establishing a causal relationship between weight gain and cognitive function. The aim of this longitudinal study is to examine the relationship between weight gain and cognitive function in patients with first-episode schizophrenia (FES) during the initial 6-month antipsychotic treatments. Employing linear and logistic regression analyses, the study involved 337 participants. Significantly, baseline scores in processing speed (OR = 0.834, *p* = 0.007), working memory and attention (OR = 0.889, *p* = 0.043), and executive function (OR = 0.862, *p* = 0.006) were associated with clinically relevant weight gain (CRW, defined as an increase in body weight > 7%) at the 6-month endpoint. On the other hand, CRW correlated with improvements in the Brief Visuospatial Memory Test (*p* = 0.037). These findings suggest that patients with lower baseline cognitive performance undergo more substantial weight gain. Conversely, weight gain was correlated with cognitive improvements, particularly in the domain of visual learning and memory. This suggested a potential bidirectional relationship between weight gain and cognitive function in first-episode schizophrenia patients.

## 1. Introduction

Schizophrenia, a severe mental illness characterized by diverse psychiatric symptoms, primarily encompasses positive symptoms such as delusions and hallucinations and negative symptoms, including apathy, anhedonia, emotional blunting, social withdrawal, and cognitive impairment. Its lifetime prevalence is estimated at around 1% worldwide [1]. Alarmingly, individuals with schizophrenia face a heightened risk of mortality, with rates 2.5 times higher than those in the general population [2]. Moreover, the condition takes a substantial toll on life expectancy, leading to a reduction of 10–20 years [3].

Individuals with schizophrenia have a significantly higher prevalence of obesity, ranging from 40% to 60%, compared to the general population [4,5,6]. In China, the obesity rate for first-episode drug-naïve schizophrenia patients is approximately 10% [7]. Inflammation resulting from obesity may play a pathogenic role in causing insulin resistance, defective insulin secretion, and disruption of energy homeostasis. Obesity-induced inflammation encompasses various organs, including fat, pancreas, liver, skeletal muscle, heart, and brain, potentially resulting in severe tissue damage [8]. Obesity contributes to an elevated risk of cardiometabolic diseases, such as type 2 diabetes mellitus, coronary artery disease, dyslipidemia, and hypertension [5,9,10], which are major factors leading to higher mortality rates and reduced life expectancy in schizophrenia [11].

Unhealthy habits such as poor diet, smoking, and inadequate exercise are prevalent among individuals with schizophrenia, elevating the risk of developing metabolic syndrome [12]. Notably, the primary side effect of second-generation antipsychotics is their propensity to induce weight gain and metabolic alterations [13]. Weight gain in schizophrenia is attributed to an interaction between lifestyle factors and treatment effects [14]. Particularly, first-episode schizophrenia (FES) patients may experience rapid and substantial weight gain in the early phase of antipsychotic treatments, highlighting the susceptibility of this population and the importance of early intervention [15,16]. However, multiple studies suggest that weight gain is associated with a favorable treatment prognosis [17]. This implies that treatment-induced weight gain may not be entirely detrimental.

Cognitive impairment is a prominent feature in schizophrenia that negatively impacts patients’ prognosis and social interactions and, therefore, contributes to the disabling nature of schizophrenia [18,19]. Most patients experience both broad cognitive impairments and impairments in specific domains, including learning, memory, attention, processing speed, and executive function [20]. Premorbid generalized cognitive impairment is identified to persist throughout the course of illness [21]. While the pathophysiological mechanisms causing cognitive deficits remain not fully understood, numerous findings suggest that dysregulation of dopaminergic, cholinergic, glutamatergic, and GABAergic systems collectively contributes to cognitive impairment. This dysregulation affects the balanced interactions between excitatory and inhibitory (E/I) neurons within cortical microcircuits [22]. Among these, dysfunction in the mesolimbic and mesocortical circuits, along with their correlative glutamatergic inputs, is believed to play a crucial role in cognitive dysfunction [23]. General evidence suggests there are relationships between cognitive deficits and negative symptoms [24]. Both neurocognitive deficits and negative symptoms contribute to the dysfunction in schizophrenia through pathways influenced by dysfunctional cognitive biases [25]. Second-generation antipsychotics are believed to slightly improve patients’ cognitive function, while none of them are found to have a satisfactory profile [26].

Emerging evidence suggests that metabolic disturbances are related to cognitive impairments [27,28]. Some studies have reported a correlation between obesity and cognitive impairments in patients suffering from schizophrenia [29,30], while others have presented contradictory results [31,32]. In a recent 12-month longitudinal study, weight gain is found to be correlated with improvements in overall cognitive function, particularly in the working memory domain, in patients with first-episode schizophrenia spectrum disorders [33]. Discrepancies in the findings of the aforementioned studies could stem from variations in sample selection and study methodology. When considering the connection between cognitive function and weight gain, distinctions may arise between patients with first-episode schizophrenia and those with chronic schizophrenia. Additionally, outcomes may diverge between cross-sectional and prospective studies.

Cognitive deficits are considered a risk factor for weight gain, as they influence the self-control in eating behaviors [34,35]. A study involving preschool children has revealed that those with higher cognitive function were less likely to become overweight [36]. Bond et al. have reported that poorer baseline cognitive function could predict weight gain over a 12-month period in patients suffered from bipolar disorder [37]. However, few studies have explored the relationship between baseline cognitive function and weight gain in patients with schizophrenia.

In summary, there is a scarcity of well-designed longitudinal studies investigating the interrelationship between cognitive function and weight gain during the initial antipsychotic treatment phase in patients with first-episode schizophrenia. In response to this knowledge gap, we conducted a second analysis of the data from the Chinese First-Episode Schizophrenia Trial (CNFEST) to examine the relationship between weight gain and cognitive function over the first 6-month antipsychotic treatments. We hypothesized that baseline cognitive impairments might be linked to weight gain during treatment, and conversely, weight gain might be associated with changes in cognitive function as well.

## 2. Materials and Methods

### 2.1. Participants and Setting

CNFEST was a multicenter, randomized clinical trial conducted in several psychiatric hospitals nationwide from 2008 to 2010 [38]. The trial was registered on ClinicalTrials.gov (No. NCT01057849) and approved by the ethics committees of the Ethics Committee of Peking University Sixth Hospital. Written informed consent was obtained from all participants. Participants were 18–45 years old with a diagnosis of schizophrenia, which was confirmed by the Structured Clinical Interview for DSM-IV [39]. Their illness duration was less than three years, with continuous antipsychotic treatment less than four weeks. Participants with comorbid other major physical or mental health problems (including alcohol and substance abuse), exposed to long-acting antipsychotic injections, or contraindicated to antipsychotics were excluded. Eligible participants were randomly assigned to aripiprazole (15–30 mg/day), risperidone (3–6 mg/day), or olanzapine (10–25 mg/day) treatment group. Participants who showed little benefit from the initial treatment were allowed to undergo an antipsychotic switching after the first four weeks. Besides, oral benzhexol (2–6 mg/day), promethazine (25–75 mg/day), or lorazepam (0.5–1.5 mg/day) could be prescribed to minimize the drop-outs.

### 2.2. Assessments

Three psychiatrists who completed consistent training for all instruments conducted clinical assessments at each study center. Psychopathology was measured using the Positive and Negative Syndrome Scale (PANSS)–Chinese Version. The internal consistency reliability, indicated by Cronbach’s α, was 0.87, and the internal reliability of the raters was 0.68 [40]. The PANSS scale is a comprehensive assessment tool for schizophrenia symptoms, consisting of 30 items. It is designed to evaluate three main domains: the positive subscale (7 items), the negative subscale (7 items), and the general psychopathology subscale (14 items) [41]. As both the positive and negative subscales include some symptoms that are not relevant to the examined construct, we additionally utilized the PANSS positive factors (P1, P3, P5, P6, and G9) and negative factors (N1, N2, N3, N4, and N6) [42,43].

Three other psychiatrists who were certified by the HIV Neurobehavioral Research Center of the University of California at San Diego performed cognitive assessments at each center [44]. The neuropsychological tests were supplied by the HIV Neurobehavioral Research Center at the University of California, San Diego. Their clinical validity and test-retest reliability were verified among patients with schizophrenia and healthy controls in China, with an Intraclass Correlation Coefficient ranging from 0.73 to 0.94 [45,46]. The ten tests assessed six main cognitive function domains were administered to measure participants’ cognitive functioning at baseline (T1) and at the 6-month follow-up endpoint (T2) (see Table 1 for listing).

Body weight was measured at baseline and 6-month follow-up. Participants removed overcoats and shoes and weighed themselves on the same calibrated electronic scale. Body mass index (BMI) was calculated as body weight in kilograms (kg) divided by height in meters squared (m^2^). Clinically relevant weight gain (CRW) was defined as an increase in body weight exceeding 7% from baseline to post-treatment [47]. The criterion of weight gain exceeding 7% has been employed in various studies to evaluate the metabolic side effects of antipsychotics [48,49,50,51].

### 2.3. Statistical Analysis

Data were analyzed using SPSS software version 25.0 (IBM Corp., Armonk, NY, USA). All raw scores of cognitive tests were transformed into scale scores, with a mean of 10 and a standard deviation of 3 [52]. Scores of cognitive domains were calculated using the average scale scores of all included tests. The composite cognitive scores, which represented global cognitive function, were calculated by averaging scale scores of all cognitive tests. We performed logistic regression analyses to assess the impact of baseline cognitive function on weight gain, using CRW as the independent binary outcome. Variables displaying suggestive indications (*p* < 0.10) of association in the univariate analysis were included in logistic regression models. Odds ratios (OR) and 95% confidence intervals (CI) were used to quantify the extent of the effect. We further conducted linear regression analyses to explore the effect of CRW on changes in cognitive function, with the change rates of cognitive test scores as the dependent variables and CRW as the independent variable. To account for confounding factors, we incorporated demographic variables (gender, age, and education), treatment factors (including type and dose of antipsychotics, combined use of benzodiazepines or anticholinergics), and symptom improvements (rate of PANSS score reductions) as control variables. These variables are considered potentially associated with changes in cognitive function. Since scale scores cannot be used for comparison at different time points, raw scores of each cognitive test were used in this analysis. All statistical tests were 2-tailed, with a *p*-value less than 0.05 considered significant.

## 3. Results

### 3.1. Sample Description and Baseline Characteristics

A total of 337 participants completed both the baseline and the 6-month follow-up assessments. Among them, 115 were from the risperidone group, 114 from the olanzapine group, and 108 from the aripiprazole group. The demographic, clinical, and metabolic characteristics of study participants at baseline were presented in Table 2. No significant differences were observed between the three groups of participants.

### 3.2. Cognitive Function and Weight Changes during the First 6-Month Treatment

The raw scores of cognitive tests and body weight at baseline (T1) and the 6-month follow-up endpoint (T2) were presented in Table 3. Except for Animals-Naming and HVLT-R tests, other tests showed significant improvements during the follow-up period (all *p* < 0.05). The average weight gain was 6.3 kg during the 6-month antipsychotic treatment period, corresponding to an average rate of weight gain of 10.8%. A total of 214 patients (64.3%) experienced clinically relevant weight gain (CRW).

### 3.3. The Relationship between Baseline Cognitive Function and CRW at the Follow-Up Endpoint

When comparing the baseline parameters, potential significant differences (*p* < 0.1) were observed in several factors between participants with CRW and those without CRW. These factors include gender (*p* = 0.079), age (*p* = 0.016), baseline BMI (*p* < 0.001), treated with olanzapine (*p* = 0.001), antipsychotic dose (*p* = 0.008) domain scores in baseline processing speed (*p* = 0.034), working memory and attention (*p* = 0.084), and executive function (*p* = 0.017) (Supplementary Table S1). Therefore, we conducted logistic regression analyses with the above three baseline cognitive domain scores as independent variables and CRW as the dependent variable, controlling for age, gender, baseline BMI, treatment group, and antipsychotic dose. Our results indicated that worse performances in all three cognitive domains at baseline [processing speed (OR = 0.834, *p* = 0.007), working memory and attention (OR = 0.889, *p* = 0.043), and executive function (OR = 0.862, *p* = 0.006)] were associated with CRW at the 6-month follow-up endpoint (Table 4).

### 3.4. The relationship between CRW and Cognitive Improvements during the 6-Month Treatment

When comparing the cognitive improvements over the course of 6 months, participants who experienced CRW exhibited more improvements in BVMT-R delayed recall (*p* = 0.042) and Spatial Span tests (*p* = 0.031) (Supplementary Table S2). Therefore, we conducted linear regression analyses with the change rates of BVMT-R delayed recall and Spatial Span tests as dependent variables and CRW as the independent variable, controlling for demographic variables (such as gender, age, and education), treatment factors (including type and dose of antipsychotics, combined use of benzodiazepines or anticholinergic), and symptom improvements (rate of PANSS total, positive and negative factors scores reductions). Since there were no significant differences in baseline cognitive test scores between participants with CRW and those without CRW (*p* > 0.05), we chose not to include baseline cognitive test scores as confounding variables. CRW showed significant association with improvements in the BVMT-R delayed recall test (β = 0.122, *p* = 0.037) (adjusted-R^2^ = 0.032, F = 1.948, *p* = 0.033). A marginal association was observed with improvements in the Spatial Span test (*p* = 0.077) (Table 5).

## 4. Discussion

To the best of our knowledge, this study was one of the largest longitudinal studies investigating the relationship between weight gain and cognitive function in patients with first-episode schizophrenia. Our results revealed a bidirectional relationship. On one hand, poorer baseline cognitive performance, especially in the processing speed, working memory and attention, and executive function domains, were related to clinically relevant weight gain (CRW) over the first 6-month antipsychotic treatment. On the other hand, CRW was associated with cognitive improvements in the visual learning and memory domain, which was independent of psychopathology improvement.

Our results demonstrated significant improvements in nearly all cognitive function domains together with significant weight gain during the first 6-month treatment in patients with first-episode schizophrenia, which were consistent with previous studies [53,54].

We found that poorer baseline performance in processing speed, working memory and attention, and executive function were correlated with clinically relevant weight gain during the 6-month treatment period. Previous studies conducted in healthy populations and patients with bipolar disorder also have reported unanimous correlation [37,55]. Similarly, Jakobsen et al. have revealed that higher baseline cognitive function might predict a more advantageous metabolic profile in patients with schizophrenia spectrum disorders [56]. The effect of cognitive function on weight gain may be mediated by eating behavior [57,58]. People with schizophrenia have been observed to exhibit significantly unhealthy dietary habits [59]. Disordered eating behaviors are believed to be associated with impaired frontal lobe function [60], which is prominent in the pathophysiology and cognitive impairment of schizophrenia [61,62]. Individuals with lower working memory tend to choose less healthy foods and face a higher likelihood of failure in dietary interventions [63]. In contrast, those with superior working memory experience quicker satisfaction from stimuli [64]. Conversely, individuals with poor executive function often struggle with delaying gratification, leading to ineffective restraint in eating behaviors [65].

Metabolic abnormalities in first-episode schizophrenia are not solely attributed to antipsychotic treatment but are intrinsic to the disorder itself. Components of the metabolic syndrome have been observed in first-episode drug-naïve early-onset schizophrenia. Despite the average age of this patient group being less than 18 years old, nearly 10% of them were identified as having metabolic syndrome [66]. A meta-analysis has uncovered that drug-naïve patients experiencing a first episode of psychosis (FEP) exhibit elevated blood concentrations of adrenocorticotropic hormone (ACTH) and prolactin (PRL), along with lower levels of thyroid-stimulating hormone (TSH) [67]. These anterior pituitary hormones play a crucial role in energy metabolism and weight regulation [68]. Moreover, genome-wide association studies (GWAS) have revealed potential shared genetic variations or pathways between schizophrenia and metabolic abnormalities [69]. In conjunction with the current study’s revelation of the association between cognitive impairment and weight gain, it implies that cognitive dysfunction and metabolic abnormalities might share a common pathological basis in schizophrenia.

Research evidence indicates a relationship between psychopathology improvement and weight gain induced by antipsychotics [70,71]. Notably, our results demonstrated that the correlation between weight gain and cognitive enhancement persisted after accounting for psychotic symptom improvements. We proposed several possible explanations for this result. Firstly, 5-HT receptors, which are targets for atypical antipsychotics, are involved in both cognitive function and weight gain [72,73]. Secondly, the physiological effects of insulin in the central nervous system include the regulation of striatal dopamine levels, peripheral glucose homeostasis, body weight, and cognitive performance [74]. Thus, insulin regulation may play a role in the correlation between cognitive improvements and weight gain [75]. Thirdly, the composition of gut microbiota is linked to cognitive impairment in schizophrenia [76]. Furthermore, changes in the gut microbiome observed in individuals on antipsychotic medications may be connected to weight gain [77]. Notably, alterations in gut microbiota could potentially mediate the relationship between weight gain and cognitive function. Fourthly, patients with better treatment compliance experience greater improvements in cognitive function during treatment [48]. Consequently, individuals with better treatment compliance may also receive higher doses of antipsychotics or undergo longer treatment durations, potentially leading to increased antipsychotic-induced weight gain [78].

Several studies have reported an association between obesity and compromised cognitive function [79,80], which is inconsistent with our results. Contradictory evidence exists as well. Earlier studies have identified positive correlations between blood glucose levels and the domains of working memory and attention/vigilance, along with positive correlations between total cholesterol levels and social cognition in first-episode drug-naïve patients [81,82]. These findings suggest that abnormalities in specific metabolic profiles might actually be protective of cognitive functioning in the early stages of the disease, which aligns with our results. The association between various metabolic markers and cognitive function seems inconsistent across different stages of the disease. However, all the aforementioned studies are cross-sectional, preventing the investigation of the longitudinal relationship between weight gain and cognitive changes. We found only one prospective cohort study in this field. Luckhoff et al. discovered that as their BMI increased, patients with first-episode schizophrenia spectrum disorders exhibited improved working memory function and overall cognitive function over a 12-month treatment period [33], aligning with our findings. However, it is crucial to note that this correlation may change as the disease progresses and treatment persists [83]. It is plausible that the adverse effects of metabolic syndrome accumulate to a threshold, ultimately resulting in cognitive impairments [84].

Our findings have considerable clinical implications. Dietary interventions are effective in reducing weight and improving cognitive function in the obese population [85]. Previous research indicates that a dietary plan with reduced calorie intake can improve cognitive function and reduce metabolic indicators in patients with schizophrenia [86]. Additionally, there is evidence suggesting that increasing aerobic exercise can effectively enhance cognitive function in patients with schizophrenia [87]. Cognitive behavioral therapy is beneficial in managing weight gain in patients treated with antipsychotics [88]. Thus, we recommend early weight management and cognitive remediation, as they might offer dual benefits for patients with schizophrenia. Healthcare providers should raise awareness about the bidirectional relationship between weight gain and cognitive function and provide appropriate intervention recommendations for patients and their family members. By implementing effective interventions targeting both weight management and cognitive function, healthcare providers can contribute to enhancing the overall well-being and quality of life of patients with schizophrenia [89].

This study had several strengths which contributed to its scientific rigor. The utilization of a longitudinal design, detailed cognitive assessments, and a relatively large sample size enhanced the validity of our findings. Focusing on minimally treated first-episode schizophrenia patients allowed us to control confounding factors, including illness duration and complex medication regimens. Nevertheless, several warrant limitations should be taken into consideration. Firstly, the relatively short follow-up duration and the somewhat high drop-out rate might limit our ability to observe the long-term relationship between weight gain and cognitive function. Secondly, we did not include laboratory metabolic indicators, such as blood pressure, glucose level, and lipids profile, which might restrict our ability to provide more objective results. Thirdly, psychosocial factors such as dietary habits and physical activities were not collected in the original study, potentially impacting the relationship between weight change and cognitive function. Fourthly, in this study, we utilized a linear regression model for variable screening instead of approaches that consider the weight of individual components, such as a LASSO (Least Absolute Shrinkage and Selection Operator) model. This choice may affect the external validity and interpretability of the model.

Future studies should evaluate cognitive function in a sample of well-characterized first-episode drug-naïve schizophrenia patients utilizing a comprehensive set of neurocognitive test batteries for schizophrenia, such as The MATRICS Consensus Cognitive Battery (MCCB). Additionally, the integration of metabolic biochemical and brain imaging indices, along with the incorporation of lifestyle factors, is essential to explore the pathological mechanism underlying the relationship between metabolic syndrome and cognitive function. Further investigation should consider the evolving dynamics of this relationship over an extended follow-up period.

## 5. Conclusions

In conclusion, our study suggested that patients in their first episode of schizophrenia, with poorer baseline cognitive performance, particularly in the domains of processing speed, working memory and attention, and executive function, tended to experience more prominent weight gain during the first 6-month antipsychotic treatment. Intriguingly, weight gain was found to be associated with cognitive improvements in the visual learning and memory domain. These findings implied a bidirectional relationship between weight gain and cognitive function, suggesting a connection that extends beyond a simple causal link. The present study addressed the lack of large-sample longitudinal studies in this research field. It produced findings that deviate from the majority of previous cross-sectional studies, indicating that weight gain during the initial phase of schizophrenia treatment had a beneficial effect on cognitive function. Furthermore, this study validates that cognitive impairment served as a risk factor for weight gain, a pattern observed in both healthy populations and those with other mental disorders. Our research shed new light on further understanding the relationship between metabolic syndrome and cognitive function in schizophrenia. Further study should delve into the association between metabolic syndrome and cognitive function within the specific context of schizophrenia, considering both its nature and progression.

## Figures and Tables

**Table 1:** Cognitive test list.

Cognitive Domains	Tests
Processing speed	Trail Making Test Part A
	Color Trails Test 1
	Animals-Naming
	Stroop Color and Word Test
Vocabulary learning and memory	Hopkins Verbal Learning Test-Revised
Visual learning and memory	Brief Visuospatial Memory Test-Revised
Working memory and attention	Wechsler Memory Scale-spatial span subtest
Executive function	Paced Auditory Serial Addition Test
	Color Trails Test 2
	Stroop Test-unconscious
Fine motor function	Grooved Pegboard Test–dominant and non-dominant hand

**Table 2:** Baseline demographic, clinical, and metabolic characteristics.

	Whole Sample (*N* = 337)	Risperidone (*N* = 115)	Olanzapine (*N* = 114)	Aripiprazole (*N* = 108)	*p*
**Demographics**					
Age, mean (SD), y	25.03 (7.17)	25.81 (7.15)	24.43 (7.41)	24.82 (6.90)	0.326
Male, %	49.55	53.91	51.75	42.59	0.203
Single, %	83.48	86.08	81.57	82.69	0.634
Education years, mean (SD), y	12.55 (2.82)	12.64 (2.79)	12.59 (2.86)	12.43 (2.82)	0.846
**Clinical characteristics**					
Duration of illness, mean (SD)	10.28 (10.21)	10.50 (9.45)	10.02 (10.13)	10.33 (11.15)	0.944
PANSS Positive, mean (SD)	22.95 (5.53)	23.49 (6.20)	22.74 (5.22)	22.60 (5.07)	0.431
PANSS Negative, mean (SD)	20.67 (7.28)	20.68 (7.28)	20.53 (7.60)	20.82 (6.98)	0.956
PANSS General, mean (SD)	42.14 (8.48)	41.97 (7.53)	42.28 (9.55)	42.17 (8.30)	0.960
PANSS Total, mean (SD)	85.54 (14.60)	86.20 (14.17)	84.81 (14.73)	85.63 (15.02)	0.770
**Metabolic characteristics**					
Weight, mean (SD), kg	58.50 (11.19)	59.91 (11.86)	57.18 (10.89)	58.37 (10.67)	0.185
BMI, mean (SD), kg/m^2^	21.05 (3.07)	21.35 (2.97)	20.72 (3.07)	21.08 (3.16)	0.305

PANSS: Positive and Negative Syndrome Scale.

**Table 3:** Comparisons of cognitive tests raw scores and body weight at baseline (T1) and at the 6-month follow-up endpoint (T2).

	Baseline Mean (SD)	6-Month Follow-Up Mean (SD)	t	*p*
**Processing speed**				
TRAIL A	49.82 (23.26)	40.01 (16.77)	9.613	<0.001
CTT1	57.21 (29.34)	46.89 (27.64)	6.764	<0.001
Animals-Naming	17.02 (5.58)	17.00 (5.58)	0.085	0.932
Stroop Word	79.39 (21.30)	82.43 (18.18)	−3.221	0.001
Stroop Color	54.00 (16.91)	56.58 (14.48)	−3.734	<0.001
**Vocabulary learning and memory**				
HVLT-R Learning (total 3 trials)	22.16 (6.41)	22.52 (5.35)	−1.054	0.293
HVLT-R delayed recall	7.57 (2.93)	7.49 (2.70)	0.454	0.650
**Visual learning and memory**				
BVMT-R Learning (total 3 trials)	22.18 (7.50)	24.01 (6.89)	−5.039	<0.001
BVMT-R delayed recall	8.97 (2.94)	9.41 (2.45)	−2.949	0.003
**Working memory and attention**				
Spatial Span	14.98 (3.81)	16.05 (3.48)	−5.911	<0.001
PASAT	31.01 (11.00)	37.16 (9.52)	−12.162	<0.001
**Executive function**				
CTT2	119.58 (58.24)	95.63 (36.53)	8.850	<0.001
Stroop Unconscious	31.34 (11.33)	34.01 (10.06)	−5.605	<0.001
**Fine motor function**				
Peg-SD	85.46 (28.65)	77.52 (17.25)	5.860	<0.001
Peg-SN	95.02 (32.64)	90.02 (24.85)	3.164	0.002
Body Weight	58.54 (11.18)	64.89 (11.14)	−21.147	<0.001

TRAIL A: Trail Making Test Part A, CTT1: Color Trails Test 1, CTT2: Color Trails Test 2, HVLT-R: Hopkins Verbal Learning Test-Revised, BVMT-R: Brief Visuospatial Memory Test-Revised, Spatial Span: Wechsler Memory Scale-spatial span subtest, PASAT: paced auditory serial addition test, Peg-SD: Grooved Pegboard Test dominant hand, Peg-SN: Grooved Pegboard Test non-dominant hand.

**Table 4:** Logistic regression models of the relationships between baseline cognitive function and CRW.

	OR	95% CI	*p*
Model 1			
Processing speed T1	0.834	(0.731, 0.952)	**0.007**
Gender	0.701	(0.416, 1.181)	0.185
Age	0.971	(0.936, 1.008)	0.129
BMI T1	0.769	(0.701, 0.843)	**<0.001**
Treated with olanzapine	3.097	(1.509, 6.357)	**<0.001**
Antipsychotic dose (in olanzapine equivalent)	0.991	(0.938, 1.048)	0.756
Model 2			
Working memory and attention T1	0.889	(0.794, 0.996)	**0.043**
Gender	0.771	(0.462, 1.285)	0.318
Age	0.985	(0.951, 1.021)	0.402
BMI T1	0.769	(0.701, 0.844)	**<0.001**
Treated with olanzapine	2.869	(1.410, 5.839)	**0.004**
Antipsychotic dose (in olanzapine equivalent)	0.999	(0.946, 1.055)	0.978
Model 3			
Executive function T1	0.862	(0.776, 0.958)	**0.006**
Gender	0.724	(0.430, 1.217)	0.223
Age	0.969	(0.933, 1.006)	0.099
BMI T1	0.767	(0.699, 0.843)	**<0.001**
Treated with olanzapine	2.882	(1.423, 5.838)	**0.003**
Antipsychotic dose (in olanzapine equivalent)	0.998	(0.945, 1.054)	0.936

T1: baseline, BMI: Body Mass Index. Statistically significant results (*p* < 0.05) are showed in bold.

**Table 5:** (a) Linear regression model of the relationship between CRW and improvement in BVMT-R delayed recall test during 6-month treatment. (b) Linear regression model of the relationship between CRW and improvement in Spatial Span test during 6-month treatment.

**(a)**			
**ΔBVMT-R Delayed Recall**	**Standardized Beta-Coefficient**	**T**	***p***
CRW	0.122	2.090	**0.037**
Gender	0.082	1.453	0.147
Age	−0.013	−0.231	0.817
Education years	−0.071	−1.252	0.212
ΔPANSS Positive Factor scores	0.097	1.235	0.218
ΔPANSS Negative Factor scores	0.210	2.474	**0.014**
ΔPANSS Total scores	0.301	2.852	**0.004**
Treatment groups	0.129	1.296	0.196
Antipsychotic dose (in olanzapine equivalent)	0.120	1.377	0.170
Combined use of benzodiazepines or anticholinergics	−0.016	−0.271	0.787
BVMT-R: Brief Visuospatial Memory Test-Revised, CRW: clinically relevant weight gain, PANSS: Positive and Negative Syndrome Scale. Δ was calculated by (T2 scores−T1 scores)/T1 scores. Statistically significant results (*p* < 0.05) are showed in bold.			
**(b)**			
**ΔSpatial Span**	**Standardized Beta-Coefficient**	**T**	***p***
CRW	0.105	1.777	0.077
Gender	−0.010	−0.173	0.863
Age	0.092	1.587	0.114
Education years	−0.064	−1.111	0.268
ΔPANSS Positive Factor scores	0.059	0.733	0.464
ΔPANSS Negative Factor scores	0.073	0.848	0.397
ΔPANSS Total scores	0.050	0.468	0.640
Treatment groups	0.035	0.352	0.725
Antipsychotic dose (in olanzapine equivalent)	0.043	0.491	0.623
Combined use of benzodiazepines or anticholinergics	−0.069	−1.131	0.259
Spatial Span: Wechsler Memory Scale-spatial span subtest, CRW: clinically relevant weight gain, PANSS: Positive and Negative Syndrome Scale. Δ was calculated by (T2 scores−T1 scores)/T1 scores.			

## Data Availability

The datasets presented in this article are not readily available because the data are part of an ongoing study. Requests to access the datasets should be directed to the corresponding author, Xin Yu.

## Supplementary Materials

The following supporting information can be downloaded at: https://www.mdpi.com/article/10.3390/brainsci14040310/s1, Table S1: Comparisons of baseline clinical and sociodemographic characteristics between CRW and Non-CRW group; Table S2: Comparison of cognitive improvements between participants with CRW and without CRW.
