# Supplementary material for: The Bidirectional Relationship between Weight Gain and Cognitive Function in First-Episode Schizophrenia: A Longitudinal Study in China
Source: Brain Sci. 2024 Mar 26;14(4):310. doi: 10.3390/brainsci14040310 (PMC11048552; doi:10.3390/brainsci14040310)
Supplement: Supplementary file 1 [file brainsci-14-00310-s001.zip › brainsci-2910384-supplementary.pdf]

Supplementary Material

Supplementary Tables

Table S1. Comparisons of baseline clinical and sociodemographic characteristics between CRW and Non-CRW group.

	**Non-CRW Mean (SD) (N=119)**	**CRW Mean (SD) (N=214)**	χ^2	**t**	***p***
Gender: Male, %	56.3	46.3	3.084		**0.079**
Age	26.28 (6.82)	24.32 (7.23)		2.418	**0.016**
Education years	12.82 (2.87)	12.34 (2.77)		1.498	0.135
BMI T1	22.54 (3.12)	20.22 (2.71)		7.080	**<0.001**
Risperidone, %	39.47	60.52			
Olanzapine, %	22.32	77.68			
Aripiprazole, %	45.79	54.21	14.183		**0.001**
Antipsychotic dose (in olanzapine equivalent)	13.80 (5.83)	15.68 (6.18)		-2.686	**0.008**
Combined use of benzodiazepines or anticholinergics, %	44.54	52.34	1.861		0.173
PANSS Positive score T1	23.25 (5.51)	23.00 (5.35)		0.415	0.678
PANSS Negative score T1	21.10 (7.07)	20.50 (7.40)		0.718	0.473
PANSS General score T1	42.21(7.73)	42.22 (8.91)		-0.006	0.995
PANSS Total score T1	86.56 (14.78)	85.38 (14.34)		0.715	0.475
Processing speed T1	10.33 (2.00)	9.79 (2.31)		2.125	**0.034**
Vocabulary learning and memory T1	10.13 (2.70)	9.83 (2.75)		0.945	0.345
Visual learning and memory T1	10.01 (2.55)	9.96 (2.82)		0.245	0.807
Working memory and attention T1	10.27 (2.36)	9.79 (2.45)		1.735	**0.084**
Executive function T1	10.38 (2.12)	9.72 (2.82)		2.408	**0.017**
Fine motor function T1	10.00 (2.85)	9.96 (2.83)		0.122	0.903
Cognitive Composite scores T1	10.19 (1.80)	9.84 (2.45)		1.644	0.101

T1: baseline, *T2:* 6-month end point, *CRW:* clinically relevant weight gain, *Non-CRW*: did not achieve clinically relevant weight gain, *BMI*: Body Mass Index, *PANSS:* Positive and Negative Syndrome Scale.

Table S2. Comparison of cognitive improvements between participants with CRW and without CRW.

Cognitive improvements	CRW, % Mean (SD) (N=214)	Non-CRW, % Mean (SD) (N=119)	t	*p*
**Processing speed**				
∆TRAIL A	-15.26 (30.24)	-7.68 (44.45)	1.838	0.067
∆CTT1	-12.84 (47.97)	-9.32 (33.73)	0.706	0.481
∆Animals-Naming	5.53 (40.75)	8.52 (38.53)	0.652	0.515
∆Stroop Word	7.61 (30.80)	8.87 (35.60)	0.336	0.737
∆Stroop Color	11.55 (36.05)	10.64 (31.97)	-0.226	0.821
**Vocabulary learning and memory**				
∆HVLT-R Learning	8.70 (44.79)	8.67 (36.57)	-0.015	0.988
∆HVLT-R Delayed Recall	11.83 (79.39)	4.34 (57.36)	-0.898	0.370
**Visual learning and memory**				
∆BVMT-R Learning	19.78 (64.27)	16.33 (43.42)	-0.522	0.602
∆BVMT-R Delayed Recall	18.71 (60.36)	7.75 (36.90)	-2.046	**0.042**
**Working memory and attention**				
∆Spatial Span	15.68 (43.47)	7.61 (24.03)	-2.171	**0.031**
∆PASAT	43.08 (120.60)	37.74 (139.91)	-0.356	0.722
**Executive function**				
∆CTT2	-12.60 (29.50)	-13.11 (28.21)	-0.153	0.879
∆Stroop Unconscious	21.36 (59.74)	14.15 (36.32)	-1.184	0.237
**Fine motor function**				
∆Peg-SD	-4.21 (21.56)	-7.93 (17.98)	-1.595	0.112
∆Peg-SN	-0.22 (24.19)	-3.55 (21.63)	-1.242	0.215

TRAIL A: Trail Making Test Part A, *CTT1*: Color Trails Test 1, *CTT2*: Color Trails Test 2, *HVLT-R*: Hopkins Verbal Learning Test-Revised, *BVMT-R*: Brief Visuospatial Memory Test-Revised, *Spatial Span*: Wechsler Memory Scale-spatial span subtest, *PASAT*: paced auditory serial addition test, *Peg-SD*: Grooved Pegboard Test dominant hand, *Peg-SN*: Grooved Pegboard Test non-dominant hand.∆ was calculated by (T2 scores-T1 scores)/T1 scores.
